# NEUROD2 Regulates *Stim1* Expression and Store-Operated Calcium Entry in Cortical Neurons

**DOI:** 10.1523/ENEURO.0255-16.2017

**Published:** 2017-03-09

**Authors:** Gokhan Guner, Gizem Guzelsoy, Fatma Sadife Isleyen, Gulcan Semra Sahin, Cansu Akkaya, Efil Bayam, Eser Ilgin Kotan, Alkan Kabakcioglu, Gulayse Ince-Dunn

**Affiliations:** 1 Molecular Biology and Genetics Department, Koç University, Istanbul 34450, Turkey; 2 Physics Department, Koç University, Istanbul 34450, Turkey

**Keywords:** calcium, genomics, Neurod2, store-operated calcium entry, transcription factor

## Abstract

Calcium signaling controls many key processes in neurons, including gene expression, axon guidance, and synaptic plasticity. In contrast to calcium influx through voltage- or neurotransmitter-gated channels, regulatory pathways that control store-operated calcium entry (SOCE) in neurons are poorly understood. Here, we report a transcriptional control of *Stim1* (stromal interaction molecule 1) gene, which is a major sensor of endoplasmic reticulum (ER) calcium levels and a regulator of SOCE. By using a genome-wide chromatin immunoprecipitation and sequencing approach in mice, we find that NEUROD2, a neurogenic transcription factor, binds to an intronic element within the *Stim1* gene. We show that NEUROD2 limits *Stim1* expression in cortical neurons and consequently fine-tunes the SOCE response upon depletion of ER calcium. Our findings reveal a novel mechanism that regulates neuronal calcium homeostasis during cortical development.

## Significance Statement

Store-operated calcium entry (SOCE) is a major source of neuronal calcium influx. Although SOCE controls key neurodevelopmental processes, the gene expression programs that regulate this mode of calcium entry in neurons remain poorly understood. In this study, we conducted an *in vivo*, genome-wide target gene analysis of the neurogenic transcription factor NEUROD2. We find that NEUROD2 controls the *Stim1* gene, which encodes a major ER calcium sensor and an essential component of SOCE. Importantly, we demonstrate that NEUROD2 is a critical regulator of neuronal SOCE levels. Our findings present important implications for understanding transcriptional programs that control neuronal calcium homeostasis, as well as for disease mechanisms in which deranged SOCE is observed, such as epilepsy and Alzheimer’s disease.

## Introduction

By a remarkable series of regulated gene expression programs, neural progenitor cells, and eventually neurons, steadily transition from one cellular state to the next in terms of their proliferative capacities, migratory behavior, axonal growth, and dendritogenic and synaptogenic capabilities (Kohwi and Doe, 2013; Pataskar et al., 2016; Telley et al., 2016). This series of regulated transitions depends on the correct spatiotemporal expression of critical transcription factors (TFs) that allow the generation of different classes of mature neurons at the correct time and place (Leone et al., 2008; Kwan et al., 2012; Greig et al., 2013). While the phenotypes emerging from knockout mouse models of these TFs have been extensively analyzed, their genome-wide binding sites and the biological implications of such binding events are still largely unknown.

Proneural and neurogenic basic helix-loop-helix (bHLH) TFs are key players for controlling the emergence of a wide range of neuronal subtypes each with unique connectivities, and physiologic and morphologic properties (Mattar et al., 2008; Wilkinson et al., 2013; Imayoshi and Kageyama, 2014). In the developing mammalian neocortex, these TFs include proneural Neurogenins (*Neurog1/2*) and neurogenic NeuroDs (*Neurod1/2/4/6*), for which ectopic gain of function is sufficient to induce neurogenesis (Farah et al., 2000; Noda et al., 2006) and for which loss-of-function mutations result in a wide-range of neurodevelopmental abnormalities (Wilkinson et al., 2013; Yuan and Hassan, 2014). At the onset of mouse cortical neurogenesis, a transient burst of expression of Neurogenins specifies neural progenitors into cortical excitatory neurons (Fode et al., 2000; Mattar et al., 2008; Kim et al., 2011). Specifically, the Neurogenins trigger a cascade of downstream TF expression, including the NeuroD family, which then simultaneously activate genetic programs to drive excitatory neurogenesis and suppress alternative cell fates (Jo et al., 2007; Roybon et al., 2010; Pataskar et al., 2016). The importance of the Neurogenin–NeuroD gene regulatory network for human neocortical development is evidenced by a recent study demonstrating that, during the evolution of the human fetal neocortex, neural progenitor populations expressing *Neurog2* had specifically expanded relative to other progenitor classes (Johnson et al., 2015). In fact, the *Neurog1/2*-driven network is being exploited for the *in vitro* production of cortical excitatory neurons from human induced pluripotent stem cells, aided by remarkable developments in cellular reprogramming and high-throughput gene expression technologies (Busskamp et al., 2014).

NEUROD2 is one of the key members of the Neurogenin–NeuroD gene network. Within the neocortex, *Neurod2* expression is triggered as progenitors exit the cell cycle and is sustained throughout the lifetime of cortical excitatory neurons (McCormick et al., 1996; Olson et al., 2001). *Neurod2* regulates several essential features of brain development, as mice lacking *Neurod2* exhibit morphologic and physiologic defects in thalamocortical connections, hippocampal synaptogenesis, axonal guidance of callosal axons, and development of amygdalar nuclei (Olson et al., 2001; Lin et al., 2005; Ince-Dunn et al., 2006; Wilke et al., 2012; Bormuth et al., 2013; Chen et al., 2016). In gain-of-function experiments, the overexpression of *Neurod2* in cortical neural progenitors induces premature exit from the cell cycle and differentiation (Telley et al., 2016). These studies clearly reveal that NEUROD2 controls a wide-range of neurodevelopmental and physiologic processes in different developmental stages and brain regions. In fact, recent target gene analyses and gene expression studies have suggested that NEUROD2 regulates components of radial migration and neuritogenesis during embryonic development (Bayam et al., 2015; Telley et al., 2016). However, questions remain regarding the genome-wide binding sites of NEUROD2 at various spatiotemporal settings and the biologically relevant effects of such binding events.

In this study, we performed a chromatin immunoprecipitation and sequencing (ChIP-Seq) analysis of NEUROD2 from postnatal cerebral cortical tissue, with the goal of identifying target genes and pathways regulating processes important for postnatal cortical development. Our *in vivo* analysis identified *Stim1* (stromal interaction molecule 1) as a primary target of NEUROD2. *Stim1* encodes a major sensor of endoplasmic reticulum (ER) calcium levels and is an important regulator of store-operated calcium entry (SOCE; Kraft, 2015; Moccia et al., 2015). Contrary to previous research describing NEUROD2 as a transcriptional activator, our data suggest that NEUROD2 restrains *Stim1* expression via binding to an intronic element within intron 2 of *Stim1*. The NEUROD2 binding site is phylogenetically conserved and harbors clustered consensus E-box elements. Knockdown of *Neurod2* expression in cultured cortical neurons increased STIM1 protein expression and consequently caused an upregulation in SOCE. Conversely, *Neurod2* overexpression resulted in depression of SOCE response. Collectively, our data point to a NEUROD2-dependent gene regulatory mechanism that controls neuronal SOCE via fine-tuning STIM1 abundance.

## Materials and Methods

### Chromatin immunoprecipitation and sequencing

Cortices were recovered from five littermate BALB/c postnatal day 0 (P0) mice of either sex. Cortical tissue was dissected, pooled, and cross-linked for 10 min in 1% formaldehyde. Cross-linked tissue was lysed in RIPA buffer (0.05 m Tris-HCl, pH 7.5, 0.15 m NaCl, 1% Triton X-100, 1% Na-DOC, 0.1% SDS) and sonicated to achieve 200–250 bp fragments. Ten percent of the input was used to isolate input chromatin, and the remainder was used for ChIP. NEUROD2–chromatin complexes were immunoprecipitated using three separate antibodies (ab168932, ab104430, and ab109406, Abcam). Chromatin immunoprecipitated with an unrelated GFP antibody was used as a negative control (sc-8334, Santa Cruz Biotechnology). Beads used for immunoprecipitation were carried through a series of stringent wash steps (Buffer 1: 1× PBS, 0.1% SDS, 0.5% Na-DOC, 0.5% NP-40; Buffer 2: 5× PBS, 0.1% SDS, 0.5% Na-DOC, 0.5% NP-40; Buffer 3: 15 mm Tris-HCl, pH 7.5, 5 mm EDTA, 2.5 mm EGTA, 1% Triton X-100, 1% Na-DOC, 0.1% SDS, 120 mm NaCl, 25 mm KCl; Buffer 4: 15 mm Tris-HCl, pH 7.5, 5 mm EDTA, 2.5 mm EGTA, 1% Triton X-100, 1% Na-DOC, 0.1% SDS, 1M NaCl; Buffer 5: 15 mm Tris-HCl, pH 7.5, 5 mm EDTA; Buffer 6: 50 mm Tris-HCl, pH 7.5, 150 mm NaCl, 1 mm MgCl_2_, 0.05% NP-40; Buffer 7: 50 mm Tris-HCl, pH 7.5, 10 mm MgCl_2_, 0.5% NP-40). After the washes, protein–DNA crosslinks were reversed at 65°C, RNase A and proteinase K treatments were conducted, and ChIP DNA was isolated by standard phenol-chloroform extraction and ethanol precipitation. Library preparation and 50 bp single end sequencing (HiSeq 2500 platform, Illumina) were performed at Genewiz (South Plainfield, NJ).

### Bioinformatics analysis

Raw sequences from three independent NEUROD2 ChIP-Seq experiments were mapped onto the mouse genome build mm10 using *Bowtie for Illumina* (version 1.1.2; Langmead et al., 2009; Langmead and Salzberg, 2012). Peak locations were determined using *MACS* (Model-based analysis of ChIP-Seq, version 1.0.1; Zhang et al., 2008). Sequence reads from GFP ChIP-Seq were used as control files for *MACS* analysis. A *p*-value of 1 × 10^−5^ was used as cutoff value for peak calling. Analyses with *Bowtie* and *MACS* tools were conducted through the Galaxy interface (usegalaxy.org; Giardine et al., 2005). Overlapping peaks identified in all three NEUROD2 ChIP-Seq datasets were selected as high confidence binding sites, and all subsequent analyses were conducted using these binding sites. Individual *p*-values and false discovery rates (FDRs) were calculated for each of the overlapping peak regions. Processed files are available at Gene Expression Omnibus (https://www.ncbi.nlm.nih.gov/geo/) under the accession number GSE84895. Midpoints of NEUROD2 binding sites were overlapped with Ensembl annotations and labeled as intergenic, exonic, intronic, or promoter [±1000 bp of a transcription start site (TSS)]. ChIP-Seq data for various histone codes were acquired from The ENCODE Project (encodeproject.org; ENCODE Project Consortium, 2012). All histone data were generated by the Bing Ren Laboratory at the University of California, San Diego (San Diego, CA), and accession numbers are as follows: H3K9me3 (ENCFF676DBG); H3K27me3 (ENCFF102IIL); H3K27ac (ENCFF145FVU); H3K36me3 (ENCFF091JOV); H3K4me3 (ENCFF875CQU); and H3K4me1 (ENCFF152TUF). CCCTC-binding factor (CTCF) ChIP-Seq data were generated by the Richard Myers Laboratory at HudsonAlpha Institute of Biotechnology (Huntsville, AL), and the accession number is: CTCF (ENCSR677HXC). Histone-binding locations and scores obtained from the ENCODE database were overlapped with NEUROD2 binding regions, yielding six histone binding scores (one for each histone type) for each NEUROD2 binding region. Mean histone-binding scores were calculated separately for NEUROD2 binding regions located in promoters, introns, exons, and intergenic regions. The genome-wide average of the histone scores was plotted as a baseline control. Gene ontology (GO) analysis was conducted as described previously (Ashburner et al., 2000). All ChIP-Seq data were visualized either in Trackster (Goecks et al., 2012) embedded in The Galaxy Project (Giardine et al., 2005) or The UCSC Genome Browser (Kent et al., 2002).

### Data access

Raw ChIP-Seq data and processed files are available at Gene Expression Omnibus (http://www.ncbi.nlm.nih.gov/geo/) under the accession number GSE84895.

### ChIP followed by quantitative PCR (ChIP-qPCR)

Ten percent of the cross-linked lysate was used to prepare input DNA by standard phenol-chloroform extraction followed by ethanol precipitation. Input and ChIP DNA were dissolved in 300 μl of sterile water, and 1 μl from each was used as template for quantitative PCR (qPCR). The adjusted input Ct value (representing 10% of input chromatin) was calculated by subtracting 3.32 (log_2_10) from the input Ct value. The following formula was then used to calculate the input normalized ChIP DNA amount: 100 × 2^(Adjusted input Ct − ChIP Ct)^. The normalized NEUROD2 ChIP DNA amounts were divided by normalized GFP ChIP DNA. The primer sequences used in ChIP-qPCR experiments were as follows: Stim1-int2-ChIP-F (gtcctgctgctgactatgtg); Stim1-int2-ChIP-R (ctaaccctttgcccctaacc); Stim1-int1-ChIP-F (gaagttctttcgtgtagtagtcatgc); Stim1-int1-ChIP-R (cagaaaggcacacctgaacaccaag); Stim1-int3-ChIP-F (aggaagggaacctcttagacaactcag); Stim1-int3-ChIP-R (ggcagtagagatggttcagtggttaag); Dlx2-ChIP-F (gacggttgcctcctttcttg); Dlx2-ChIP-R (gtcgagtgcatatcagccac); Gsx2-ChIP-F (caaaagccagttctctcccg); Gsx2-ChIP-R (ggctggtgatggtgatgatg); Gad1-ChIP-F (ccagggatcgtgcaagcaa); Gad1-ChIP-R (gtggtcttggggtctctacg); Calb2-ChIP-F (atgcgggtaggtatgcttcg); Calb2-ChIP-R (cagggcgttagcttgaagga); Npy-ChIP-F (tcacttgctggactcaggttc); Npy-ChIP-R (atgcaatctgggttcctggt); Neurod6-ChIP-F (aacagttgcaccattggcag); Neurod6-ChIP-R (gcactgatcatctggcatcc); Bhlhe22-ChIP-F (gccacacatgtcaagctaaag); Bhlhe22-ChIP-R (gccgcgagtctgaatagtttc); Nrcam-ChIP-F (aagcttcggaaacacgcac); Nrcam-ChIP-R (ggctccttgttctgctccag); and Cux1-ChIP-F (ggtgaccgatagcttgcatc); Cux1-ChIP-R (agtctccttacagtccagcg).

### Cloning

shND2-1, shND2-2, and nonsilencing (NS) short hairpin RNAs (shRNA) were cloned into pSUPER-neo-EGFP (www.oligoengine.com) as described in the manual. For calcium imaging and immunofluorescent staining experiments, the EGFP cassette was removed, and an mCherry-expressing cassette was subcloned into the *Age*I and *Not*I sites of pSUPER-neo. shND2-1-resistant cDNA (resND2) was created by site-directed mutagenesis in the pcDNA4 backbone vector by introducing three silent mutations (C1077A, G1080A, T1083A) within the shND2-1 target sequence (aagacaagagattctcgga). The primer sequences were as follows: shND2-1_F (gatccccaagacaagagattctcggattcaagagatccgagaatctcttgtcttttttta); shND2-1_R (agcttaaaaaaagacaagagattctcggatctcttgaatccgagaatctcttgtcttggg); shND2-2_F (gatcccctgccgttgagacagagcggttcaagagaccgctctgtctcaacggcattttta); shND2-2_R (agcttaaaaatgccgttgagacagagcggtctcttgaaccgctctgtctcaacggcaggg); NS_F (gatccccgcgcgatagcgctaataatttttcaagagaaaattattagcgctatcgcgcttttta); NS_R (agtctaaaaagcgcgatagcgctaataattttctcttgaaaaattattagcgctatcgcgcggg); resND2_F (ttcaccacgatcggggccccatgtac); and resND2_R (ggtgcatatcgtatgataatagattctcgga). For luciferase assays, a 570 bp fragment encompassing the NEUROD2 binding site in *Stim1* intron 2 was amplified by PCR, subcloned into the *Kpn*I and *Hind*III sites of pXPG backbone vector (Bert et al., 2000). All four E-boxes were destroyed by site-directed mutagenesis using the following primers: Stim1MutE1_F (cactcaagcaagggtccca); Stim1MutE1_R (ggtgaacagaatgtatcttccc); Stim1MutE2_F (cagacacatggagctacac); Stim1MutE2_R (ggtacacggacccttgcttg); Stim1MutE3_F (agctacacatttcagagagtaggc); Stim1MutE3_R (gattgactctgggtacacggac); Stim1MutE4_F (cctccgtcatgcttccagga); and Stim1MutE4_R (gattcacggggagctgctgcctc). All constructs created were confirmed by Sanger sequencing.

### Reverse Transcription and quantitative PCR (RT-qPCR)

Total RNA from primary cortical cultures was prepared using the Absolutely RNA Microprep Kit (Agilent Technologies) and reverse-transcribed using Transcriptor High Fidelity cDNA Synthesis Kit (Roche). The transcripts were quantified by qPCR with Luminaris HiGreen qPCR Master Mix (Thermo Scientific) using a CFX Connect Real-Time PCR Detection System (Bio-Rad). *Gapdh* RNA was used for normalization. The primer sequences were as follows: Stim1-qPCR-F (cctctcttgactcggcataatc); Stim1-qPCR-R (gaccttctctacttccacagttc); Gapdh-qPCR-F (cgacttcaacagcaactcccactcttcc); Gapdh-qPCR-R (tgggtggtccagggtttcttactcctt); Stim2-qPCR-F (ctactgtgctttcttcgccc); and Stim2-qPCR-R (aactccataccgcattgctg)

### Luciferase assays

Transfection of HEK293T cells for luciferase assay was performed in 24-well plates using Lipofectamine 2000 (Invitrogen). Each well was cotransfected with 200 ng of pXPG luciferase reporter construct (Bert et al., 2000), 200 ng of empty or NEUROD2-expressing pcDNA4 plasmid and 100 ng of Renilla pRL-null plasmid (Promega). Twenty-four hours after transfection, the cells were lysed in PLB buffer (Promega), and the activity of Firefly and Renilla luciferases were assessed using Dual-Luciferase Reporter Assay System (Promega).

### Primary cortical cultures, immunoblotting, and immunofluorescence staining

Primary cortical cultures were prepared from embryos of either sex derived from pregnant BALB/c mice on embryonic day 14.5 (E14.5). Briefly, cortices were dissected in ice-cold 1× HBSS and digested in 20 U/ml papain enzyme for 10 min at 37ºC. Digestion was terminated by treatment with 10 mg/ml trypsin inhibitor for 1 min. Tissue was triturated two to four times and plated onto plates (pre-coated with laminin and poly-d-lysine) in Basal Media Eagle supplemented with 5% FBS, 1 mm l-glutamine, penicillin/streptomycin, 1× N-2, and 1× B27 supplements (Invitrogen). For immunoblotting and RT-qPCR experiments, shRNAs were transfected by nucleofection immediately before plating (P3 primary cell 4-D nucleofector C kit, program #CU-133, Lonza). Protein lysates were collected at 5 days *in vitro* (DIV) in RIPA buffer supplemented with protease inhibitors. For immunofluorescence staining, neurons were transfected with Lipofectamine 2000 transfection reagent (Invitrogen) at 2 DIV and fixed with 4% paraformaldehyde at 5 and 8 DIV. All images were collected with Nikon 90i Eclipse confocal microscope, and analysis was performed with ImageJ software. Antibodies used were as follows: myc (sc-40, Santa Cruz Biotechnology), STIM1 (5668, Cell Signaling Technlogy), GFP (H0612, Santa Cruz Biotechnology), BETA-ACTIN (PA5-16914, ThermoFisher Scientific), NEUROD2 (ab168932, ab104430, and ab109406, Abcam), and Histone H3 (9715S, Cell Signaling Technology).

### Calcium imaging

Primary neuronal cultures were prepared from mouse E14.5 embryos as described above. At 2 DIV, pSUPER-neo-mCherry expressing either *Neurod2* shRNA or nonsilencing shRNA was transfected. For rescue experiments, resND2 in pcDNA4 backbone vector was cotransfected with Lipofectamine 2000. Calcium-imaging protocol was conducted at ∼7–9 DIV. Briefly, calcium-sensitive dye Fluo-3 dissolved in Ringer’s solution (155 mm NaCl, 10 mm d-glucose, 5 mm HEPES, 4.5 mm KCl, 2 mm CaCl_2_, and 1 mm MgCl_2_, pH 7.4) was loaded onto cells at a final concentration of 4 µm. Before imaging, cultures were treated with 1 µm TTX to prevent spontaneous activity. All live imaging experiments were performed using an XcellencePro inverted microscope (Olympus), where the imaging chamber was kept at 37°C and 5% CO_2_. ER Ca^2+^ was depleted by treatment with 5 µm thapsigargin in Ca^2+^-free Ringer’s solution. Images were acquired every 6 s for a total duration of 10 min using 40× magnification. The images were quantified with ImageJ software, based on mean signal intensity measured on neuronal somas. The quantified images were plotted as Δ*F*/*F*_o_ after background subtraction. *F*_o_ represented the minimum signal obtained in the images after background subtraction was performed. This minimum signal was set as a value of 1 and all other measurements (Δ*F*) were plotted as a ratio of this minimum signal.

### Use of animals

All animal experiments were done in accordance with guidelines provided by the Koç University, Ministries of Food, Agriculture and Live Stock, Forestry and Water Management, Turkey and the European Union. Ethics approval was obtained from the Institutional Animal Care and Use Committee of Koç University (no. 2013-1).

## Results

### ChIP-Seq reveals NEUROD2 targets in postnatal cerebral cortical tissue

**Figure 1.**
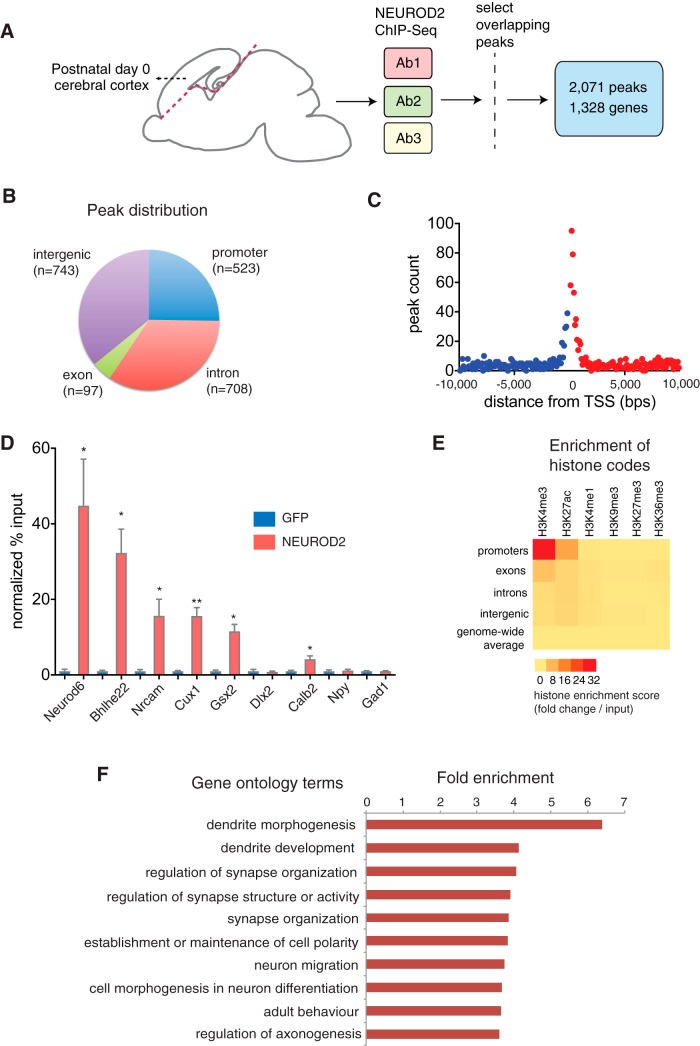
Identification of genome-wide NEUROD2 binding sites at postnatal day 0 cerebral cortex. ***A***, NEUROD2 ChIP-Seq was performed on cerebral cortex tissue using three separate antibodies. Selecting for overlapping peaks in all three datasets revealed 2,071 high confidence binding sites mapping to 1,328 annotated genes. ***B***, Distribution of midpoints of NEUROD2 binding sites based on mouse Ensembl transcripts. Midpoints mapping within ± 1000 bp of TSSs are accepted as promoter binding. ***C***, The number of NEUROD2 binding regions is plotted as a function of the distance of their midpoints to the closest TSS. A clear binding preference for NEUROD2 within ± 1000 bp is observed. ***D***, Quantification of NEUROD2 binding to target and nontarget regions by ChIP-qPCR. Template DNA is immunoprecipitated with NEUROD2 antibody or an unrelated GFP antibody as a negative control. Amount of DNA immunoprecipitated is expressed as percentage of input DNA (% input). NEUROD2 % input values are normalized to GFP % input values as described in Materials and Methods. Enrichment of NEUROD2 is detected at target regions located on *Neurod6*, *Bhlhe22*, *Nrcam*, and *Cux1* genes, but not at nontarget regions on *Dlx2*, *Npy*, and *Gad1* genes. Slight enrichment is also observed in nontarget genes *Gsx2* and *Calb2*. Bars represent SEM. *p* < 0.0001 determined by one-way ANOVA followed by unpaired *t* test, *p < 0.05, **p < 1 × 10^−4^ (Table 2). ***E***, Enrichment of histone marks within NEUROD2 peaks located within different genomic regions is represented as a heat map. Genome-wide enrichment of histone marks, including NEUROD2 target and nontarget sequences, are plotted as baseline controls. ***F***, Gene ontology analysis of all 1,328 genes identifies dendrite morphogenesis and synaptic organization as the two main NEUROD2-regulated biological processes. Significantly enriched GO categories (*p* < 0.01) are ranked based on their fold enrichment.

In the mouse, cortical neurogenesis and the process of neuronal migration largely come to an end by birth, and early postnatal days coincide with the initiation of a period of intense dendritic growth, synaptogenesis, and axonal myelination (Semple et al., 2013). Since *Neurod2* is highly expressed in the cerebral cortex during embryonic and postnatal development (Lin et al., 2004; Ince-Dunn et al., 2006) and *Neurod2* knockout mice display defects in neocortical and hippocampal dendritogenesis and synaptogenesis (Ince-Dunn et al., 2006; Wilke et al., 2012; Chen et al., 2016), we decided to identify its genome-wide targets within the cortex during this developmental time period. We dissected cerebral cortex tissue from P0 wild-type mice and conducted ChIP-Seq analysis using three separate NEUROD2 antibodies (Fig. 1*A*). We normalized our ChIP-Seq signals to a dataset acquired from a ChIP-Seq experiment conducted with an unrelated GFP antibody using the same tissue. Our computational analyses from three separate NEUROD2 ChIP-Seq experiments collectively yielded a total of 19,562 unique binding sites (*p* < 1 × 10^−5^). To minimize false-positive hits, we filtered for the 2,071 peaks that overlapped in all three experiments and represented highly significant binding regions (*p* < 1 × 10^−164^; FDR <0.004; Tables 1, Fig. 1*A*). All subsequent analyses were conducted using these 2,071 overlapping binding sites.

**Table 1:** Top 10 NEUROD2 target-binding regions

Gene symbol	Gene name	Ensembl ID	Genomic position of binding	Genomic region	Score	FDR (%)
Krtap16-1	Keratin-associated protein 16-1	ENSMUST00000105050	chr11: 99985038- 99986631	Promoter	8439.73	0
n/a	n/a	n/a	chr10: 84357827- 84358757	Intergenic	6366.16	0
Prss36	Polyserase-2 precursor	ENSMUST00000094026	chr7: 127935318- 127936794	Promoter	6362.44	0
Man1c1	Mannosidase α class 1C member 1	ENSMUST00000038628	chr4: 134579940-134581329	Exon	5669.73	0
n/a	n/a	n/a	chr12: 6638634- 6639508	Intergenic	5018.18	0
Tecr	Synaptic glycoprotein SC2 (very-long-chain enoyl-CoA reductase)	ENSMUST00000165740	chr8: 83584694-83585269	Intron	5011.04	0
Stim1	Stromal interaction molecule 1	ENSMUST00000033289	chr7: 102369602-102370206	Intron	4889.58	0
Hlcs	Holocarboxylase synthetase	ENSMUST00000163193	chr16: 94313192-94313996	Promoter	4595.15	0
Btbd17	BTB/POZ domain-containing protein 17	ENSMUST00000156192	chr11: 114795002- 114795886	Promoter	4541.48	0
Erg	Erythroblast transformation-specific transcription factor	ENSMUST00000077773	chr16: 9539059-95391295	Intron	4390.44	0

Score = −10log10(*p* value). n/a, Not applicable.

**Table 2:** Statistical table

Figure	Data structure	Type of test	Power
1*D*	Quantification of DNA by qPCR (*n* = 4–6, 2 biological replicates with each having 2–3 technical replicates)	One-way ANOVA followed by Student’s *t* test (two-tailed, type 2)	*p < 0.05, **p < 1 × 10^−4^
3*A*	Quantification of DNA by qPCR (*n* = 18, 6 biological replicates with each having 3 technical replicates)	One-way ANOVA followed by Student’s *t* test (two-tailed, type 2)	*p < 0.05, **p < 1 × 10^−4^, ***p < 1 × 10^−5^
3*B*	Measurement of luciferase reporter activity (*n* = 9, 3 biological replicates with each having 3 technical replicates)	Gaussian distribution was confirmed by D’Agostino–Pearson normality test (α = 0.05); one-way ANOVA followed by *post hoc* Tukey’s test	****p < 0.0001
4*B*	Quantification of mRNA by RT-qPCR (*n* = 9, 3 biological replicates, with each having 3 technical replicates)	Gaussian distribution was confirmed by D’Agostino–Pearson normality test (α = 0.05); one-way ANOVA followed by *post hoc* Tukey’s test	*p = 0.023
4*D*	Quantification of protein levels by immunoblotting (*n* = 3 biological replicates)	Unpaired *t* test (two-tailed, type 2)	*p* = 0.057
4*G*,*H*	Quantification of protein levels by immunofluorescence (*n* = 30, from 2 biological replicates)	Nonparametric Kruskal–Wallis test followed by Dunn’s multiple-correction analysis	*p < 0.02,**p = 0.0012, ****p < 0.0001
6*B–F*	Quantification of calcium levels by measuring Fluo-3 signal (*n* = 20–50 per condition)	Nonparametric Kruskal–Wallis test followed by Dunn’s multiple-correction analysis	**p = 0.0073, ***p = 0.0008
7*B–F*	Quantification of calcium levels by measuring Fluo-3 signal (*n* = 20–40 per condition)	Unpaired *t* test (two-tailed, type 2)	*p < 0.05

Initially, we mapped our list of genome-wide NEUROD2 binding sites onto annotated mouse transcripts from the Ensembl database (Hubbard et al., 2002). We discovered that ∼25% (*n* = 516) of all binding sites mapped onto promoter sequences, as defined by a window flanking both sides of TSSs by 1000 bp, 39% (*n* = 805) mapped onto intronic or exonic regions outside of the 2000 bp window, and another 36% (*n* = 743) mapped onto intergenic regions (Fig. 1*B*). While a majority of NEUROD2 binding sites did not map onto promoter sequences, when they were plotted relative to all TSSs in the mouse genome, a distinct binding preference was observed proximal to TSSs (Fig. 1*C*).

Next, we validated a number of selected binding sites on four different genes from our dataset, which were associated with a range of statistical confidence intervals [*Neurod6* (785.52), *Bhlhe2* (531.7), *Nrcam* (1045.99), *Cux1* (706.74); score = −10*log_10_(*p*-value)]. For quantifying NEUROD2 binding, we conducted ChIP-qPCR. As a negative control, we used template DNA, which had been chromatin-immunoprecipitated with an unrelated GFP antibody. In additional negative controls, we also performed ChIP-qPCR using primers specific to sequences on other non-target genes (*Gad1*, *Dlx2*, *Npy*, *Gsx2*, and *Calb2*). As a result, we observed a significant enrichment of NEUROD2 at all target genes tested, but not at non-target genes *Gad1*, *Dlx2*, and *Npy* (Fig. 1*D*). Unexpectedly, we also observed a low but significant enrichment at promoters of *Gsx2* and *Calb2*; whether these are biologically relevant or nonfunctional binding events remains to be elucidated.

To gain insight into the biological relevance of all 1,328 genes, which harbored NEUROD2 binding sites within their promoters or gene bodies, we conducted a GO analysis. In agreement with previous reports focused on *Neurod2* mutant phenotypes (Ince-Dunn et al., 2006; Wilke et al., 2012; Chen et al., 2016), our analysis revealed dendrite development and synapse organization as the most enriched functional categories (Fig. 1*F*). Importantly, many of the previously confirmed NEUROD2 targets, such as *Dlg4/Psd95* (Wilke et al., 2012) and *Cntn2* (Bormuth et al., 2013), as well as previously unknown targets that are key regulators of dendritogenesis and synaptogenesis, such as *Grip1*, *Nrxn3*, *Nrxn1*, and *Camk2a*, were among those genes enriched in identified GO categories.

Next, we asked whether NEUROD2 binding sites within different locations in the genome corresponded to either transcriptionally active or repressed chromatin. We acquired ChIP-Seq data collected from P0 mouse forebrain using antibodies against specific histone modifications associated with active transcription or silenced chromatin (ENCODE Project Consortium, 2012; www.encodeproject.org). Specifically, we used H3K4me3 as a marker for promoters; H3K4me1, H3K27ac and H3K36me3 as markers of active enhancers; and H3K9me3 and H3K27me3 as markers of repressed chromatin (Zhang et al., 2015). We calculated peak intensities corresponding to different histone modifications within NEUROD2 binding sequences. As a baseline value, we calculated the genome-wide average of peaks corresponding to different histone modifications (Fig. 1*E*). Our results demonstrated that NEUROD2 binding sequences mapping onto promoter regions were enriched with markers of actively transcribed promoters (H3K4me3) and enhancers (H3K4me1 and H3K27ac), (Fig. 1*E*). However, binding sites that mapped onto non-promoter gene bodies or onto intergenic regions exhibited only a low level of association with markers of active transcription or enhancers, and many NEUROD2 binding sites did not overlap with markers of either active or repressed transcription (Fig. 1*E*). Since many of the peaks mapping onto these non-promoter binding sites were among the highest ranking of all NEUROD2 peaks, we argued that at least a subset likely represented functionally relevant binding events as opposed to nonfunctional interactions (Fig. 1*B*).

### NEUROD2 binds to a conserved intronic element within the *Stim1* gene

**Figure 2.**
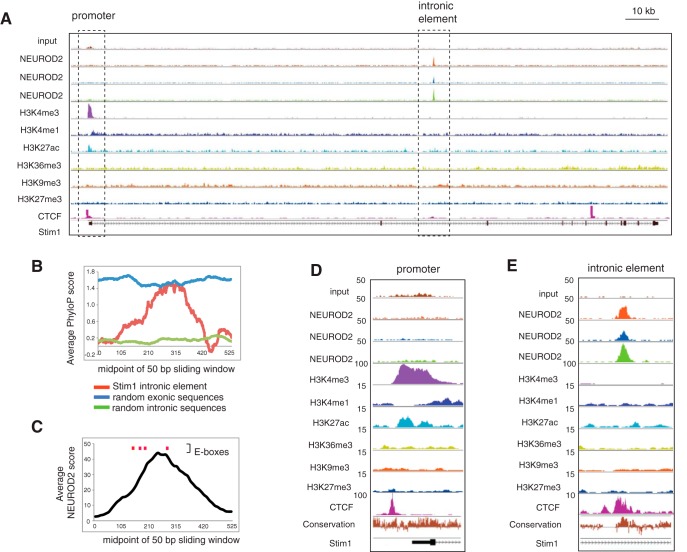
NEUROD2 binds to a conserved intronic element within the *Stim1* gene. ***A***, Input DNA or ChIP-Seq tracks acquired from three separate NEUROD2 antibodies or various histone modifications along the *Stim1* gene are plotted. ***B***, The midpoint of a 50 bp sliding window across a 550 bp stretch is plotted as a function of its average evolutionary conservation score (PhyloP score). Blue and green traces represent the average of 20 randomly selected exonic or intronic sequences within the *Stim1* gene, respectively. The red trace represents the NEUROD2 binding sequence within intron 2. ***C***, The midpoint of a 50 bp sliding window encompassing the NEUROD2 binding sequence within intron 2 is plotted as a function of NEUROD2 ChIP-Seq score (MACS score from NEUROD2 ChIP-Seq with antibody 2). Red lines denote the locations of E-boxes. ***D***, ***E***, A closer view of *Stim1* promoter and intronic element are presented. Enrichment of promoter-associated histone modifications H3K4me3 and H3K27ac are observed proximal to *Stim1* TSS. While no NEUROD2 binding is observed at the *Stim1* promoter, all three antibodies reveal strong enrichment at a specific sequence within intron 2.

Genome-wide studies of regulatory sequences have demonstrated that development and physiology of the cerebral cortex rely on the regulation of enhancer activity (Malik et al., 2014; Nord et al., 2015) and that neocortical expansion in mammals is substantially driven by changes in *cis*-regulatory elements (Reilly et al., 2015; Emera et al., 2016; Silver, 2016). However, many of the *trans*-acting factors that control the activity of these *cis*-elements and the functional outcomes of such interactions at the cellular and organismal level are largely unknown. Our observation that the majority of NEUROD2 binding sites were located outside of promoter regions suggested that at least a subset of these sites may represent important regulatory sequences that can potentially play critical roles in developmental gene expression regulation in the cortex. Therefore, next we decided to focus on how one of these potential regulatory elements was impacted by NEUROD2 binding. A binding site mapping to the second intron of the *Stim1* gene immediately attracted our attention as one of the highest scoring NEUROD2 binding sites (Table 1; Fig. 2*A*). *Stim1* gene encodes for a protein that regulates store-operated calcium entry (Liou et al., 2005). Since in the past it had been suggested that NEUROD2 is a calcium influx activated TF (Ince-Dunn et al., 2006), we decided to investigate the potential regulation of calcium influx by NEUROD2 and therefore selected *Stim1* gene for further investigation.

An examination of the *Stim1* intronic element revealed, within a 130 bp stretch, a cluster of four E-box elements (CANNTG), which is the consensus sequence for bHLH TFs like NEUROD2. We calculated the frequency of E-box distributions in all intronic sequences found in the mouse genome and found that on average one E-box is observed approximately every ∼180 bp. Therefore, four E-boxes within ∼130 bp represented approximately fivefold enrichment above random chance. Next, we examined evolutionary conservation across the *Stim1* intronic element using a tool (PhyloP) that provides a phylogenetic conservation score for each base within a target region, by comparing sequences of placental species (Cooper et al., 2005). We calculated average conservation scores within a 50 bp sliding window across a 550 bp stretch that either spanned the NEUROD2 binding site or randomly selected exonic or intronic sequences. As expected, we observed a much higher level of sequence conservation of randomly selected exons as opposed to introns (Fig. 2*B*). Surprisingly, *Stim1* intronic element also exhibited a high degree of conservation, which was comparable to random exonic sequences. Finally, we also calculated average NEUROD2 ChIP-Seq scores (*MACS* score) in a similar manner within a 50 bp sliding window across the same 550 bp stretch. As a result we observed a clear correlation between levels of NEUROD2 binding and evolutionary conservation (Fig. 2*B*,*C*).

To better understand the potential functional implications of NEUROD2 binding to *Stim1* intronic element, we asked whether this location was enriched in histone modifications that are associated with either transcriptional activation or repression. In contrast with our previous global analysis of histone modifications associated with all NEUROD2 binding sites, here we focused only on the *Stim1* gene. An analysis of peaks for different histone modifications uncovered a prominent peak for H3K4me3 (promoter marker) and less prominent but still significant peaks for H3K4me1 (enhancer marker) and H3K27ac (marker of transcriptional activity), all of which overlapped with the TSS of *Stim1* (Fig. 2*A*,*D*). Interestingly, however, the *Stim1* intronic element was not associated with histone modifications linked to promoters, enhancers, or actively transcribed or repressed chromatin (Fig. 2*A*,*E*). Moreover, NEUROD2 binding was not detected at the *Stim1* promoter, as defined by the promoter-specific histone modification H3K4me3 (Fig. 2*A*,*D*). Since this prominent NEUROD2 binding site was not associated with markers of either promoters or enhancers, next we decided to investigate the binding profile of a marker for another *cis*-regulatory element. Toward this aim, we acquired ChIP-Seq data representing binding sites of insulator protein CTCF (CCCTC-binding factor) in P0 mouse forebrain tissue (ENCODE Project Consortium, 2012; encodeproject.org). CTCF is a transcription factor commonly associated with chromatin loop formation (Hnisz et al., 2016). Evidence from recent studies have suggested that chromatin loops represent topologically associated domains, in which promoter activities are regulated by enhancers located within a loop and insulated from outside enhancers (Hnisz et al., 2016; Long et al., 2016). We observed a CTCF binding site that overlapped with NEUROD2 binding site within *Stim1* intron 2 (Fig. 2*A*,*E*). Our observation raised the possibility that NEUROD2 may function by binding to an insulator element and consequently shielding the *Stim1* promoter from the influences of inappropriate enhancer activity.

**Figure 3.**
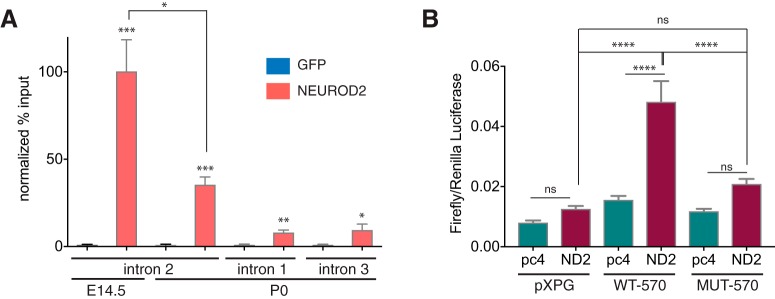
Verification of NEUROD2 binding to the conserved element within *Stim1* intron 2. ***A***, NEUROD2 binding to *Stim1* intronic element is confirmed in E14.5 and P0 cortices by ChIP-qPCR. ChIP DNA acquired with an unrelated GFP antibody is used as a negative control. Amount of DNA immunoprecipitated with either a NEUROD2 antibody (NEUROD2 ChIP DNA) or GFP antibody (GFP ChIP DNA) is expressed as percentage of input DNA (% input). NEUROD2 % input values are then normalized to GFP % input values. Strong enrichment of NEUROD2 is detected at the NEUROD2 binding element located in *Stim1* intron 2 both in E14.5 and P0 cortices. Slight enrichments are observed for *Stim1* introns 1 and 3. Data are representative of six biological replicates each composed of three technical replicates. Bars represent SEM. *p* < 0.0001 determined by one-way ANOVA followed by unpaired *t* test, *p < 0.05, **p < 1 × 10^−4^, ***p < 1 × 10^−5^ (Table 2). ***B***, Luciferase activity is measured from HEK 293T cell lysates that are transfected either with an empty luciferase reporter plasmid (pXPG) or with a luciferase reporter downstream of a wild-type (WT-570) or mutated 570 bp fragment (MUT-570) *Stim1* intronic element. In addition, cells are also cotransfected with either an empty (pc4) or NEUROD2 expressing (ND2) pcDNA4 vector. Firefly luciferase activity is normalized to Renilla luciferase signal. Data represent three independent experiments with each sample measured in triplicates. Bars represent SEM. D’Agostino–Pearson test showed normal distribution of the data (α = 0.05). One-way ANOVA and *post hoc* Tukey’s multiple-comparison analysis was performed, ****p < 0.0001 (Table 2).

Next, we confirmed NEUROD2 binding to the *Stim1* intronic element both in embryonic (E14.5) and postnatal (P0) developmental stages by ChIP-qPCR. As in our previous ChIP-qPCR analyses, as a negative control we used template chromatin DNA immunoprecipitated with a GFP antibody. In additional negative controls, we also performed ChIP-qPCR using primers specific to nontarget *Stim1* intronic sequences (introns 1 and 3). As a result, we observed a robust and highly significant enrichment of NEUROD2 at the target *Stim1* intronic element within intron 2 (100-fold for E14.5 samples and 35-fold for P0 samples; *p* < 1 × 10^−5^; Fig. 3*A*, Table 2). Notably, reduced levels of enrichment and statistical confidence were detected for sequences located on *Stim1* introns 1 and 3, most likely due to the proximity of these sequences to the strong NEUROD2 peak located on intron 2. In summary, our data support a specific binding of NEUROD2 to a conserved *Stim1* intronic element that is highly enriched in E-box elements.

Next, we tested whether the four clustered E-boxes localized to the *Stim1* intronic element were required for NEUROD2 recruitment. Toward this aim, we cloned a 570 bp fragment composed of the NEUROD2 binding site and encompassing all four E-boxes, upstream of the TSS of the luciferase gene in a reporter construct (WT-570; Bert et al., 2000). We cotransfected this reporter construct along with an empty or a NEUROD2-expressing plasmid into HEK293T cells and measured luciferase activity 24 h later. We observed that when recruited to a site immediately upstream of the TSS of a reporter gene, NEUROD2 significantly activated gene expression (Fig. 3*B*). This result was not unexpected since numerous previous studies had reported NEUROD2 acting as a transcriptional activator when bound to either endogenous promoter sequences or promoters cloned immediately upstream of reporter genes (McCormick et al., 1996; Ince-Dunn et al., 2006; Fong et al., 2012; Bayam et al., 2015). When we used a mutant reporter construct in which all four E-box elements within the 570 bp fragment were destroyed by site-directed mutagenesis (MUT-570), the capacity of NEUROD2 to influence luciferase gene expression was significantly reduced (Fig. 3*B*). These results suggested that the E-boxes are required for NEUROD2 recruitment to this particular sequence. However, given that the NEUROD2 binding site on the *Stim1* gene was located in an intron, which was far removed from the TSS and was also bound by the chromatin insulator element-associated protein CTCF, we argued that this reporter construct most likely did not faithfully recapitulate the effect of NEUROD2 on endogenous *Stim1* expression. Therefore, next we decided to investigate the effect of NEUROD2 on *Stim1* expression from its genomic locus.

### NEUROD2 limits *Stim1* gene expression in cortical neurons

**Figure 4.**
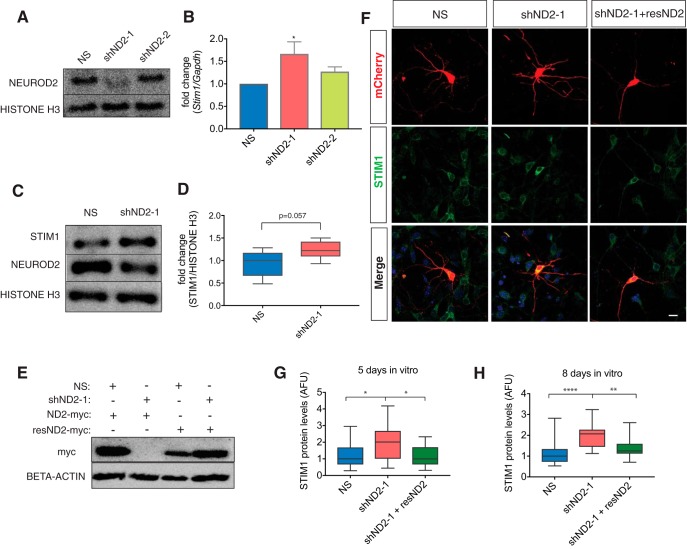
NEUROD2 suppresses *Stim1* expression. ***A***, Immunoblotting analysis reveals that two separate shRNAs (shND2-1 and shND-2) can suppress *Neurod2* expression compared with a nonsilencing shRNA (NS) in primary cortical cultures with different efficiencies. ***B***, *Neurod2* mRNA levels normalized to *Gapdh* mRNA is measured by RT-qPCR in cortical cultures transfected with either shND2-1 or shND2-2. While both shRNAs induce an upregulation of *Stim1* mRNA, the effect of the more potent shND2-1 is greater. Data represent three biological replicates, each with three technical replicates. One-way ANOVA followed by *post hoc* Tukey’s test, *p = 0.023. ***C***, ***D***, Primary cortical cultures were transfected with NS shRNA and shND2-1. STIM1 protein levels were quantified by immunoblotting and normalized to histone H3 loading control. Data are presented as bar graphs; the line marks the median; the box represents the 25th and 75th percentiles; top and bottom whiskers mark minima and maxima, respectively. Unpaired *t* test, *p* = 0.057. ***E***, resND2-myc, a cDNA resistant to shND2-1, was generated. HEK293T cells were transfected with NS shRNA or shND2-1, along with either ND2-myc or resND2-myc cDNAs. Immunoblotting analysis against the myc epitope revealed that while shND2-1 completely knocked down the expression of ND2-myc, resND2-myc expression was not affected. ***F***, Primary cortical neurons were transfected at low efficiency with shND2-1 either alone or together with resND2-myc and immunofluorescently stained against STIM1 protein. Transfected cells were identified based on their coexpression of mCherry from the shRNA-expressing plasmid. ***G***, ***H***, Quantification of STIM1 immunofluorescence signals from experiments presented in ***F***. Experimenter was blinded to all sample identity during staining and quantification. *n* = 30 for each condition from two independent experiments. Scale bar, 20 µm. Data are presented as bar graphs; the line marks the median; the box represents the 25th and 75th percentiles; top and bottom whiskers mark minima and maxima, respectively. Nonparametric Kruskal–Wallis test was followed by Dunn’s multiple-comparison analysis, *p < 0.02, **p = 0.0012, ****p < 0.0001 (Table 2).

To determine the effect of NEUROD2 on *Stim1* expression, we knocked down *Neurod2* in mouse primary cortical neurons via transfection of shRNA and quantified *Stim1* expression. To achieve a high efficiency of transfection with primary neurons, we used an electroporation-based method (nucleofection) and achieved ∼80–90% transfection efficiency. Initially, we tested two separate shRNAs and found that shND2-1 knocked down *Neurod2* expression much more efficiently compared with shND2-2 (Fig. 4*A*). Interestingly, compared with a NS shRNA, *Stim1* mRNA, and protein levels increased ∼1.3- to 1.5-fold on knockdown of *Neurod2* with the more effective shND2-1 and changed minimally with the less effective shND2-2 (Fig. 4*B–D*). Therefore, for the remaining experiments we decided to use shND2-1, which knocked down NEUROD2 levels by ∼65% of the control and subsequently resulted in aberrant *Stim1* upregulation (Fig. 4*A–D*).

Next, we questioned whether knocking down NEUROD2 expression in the majority of neurons in culture could potentially cause an unusual culture environment and result in secondary, cell-nonautonomous effects on *Stim1* expression. To eliminate this possibility, we transfected shND2-1 at a low efficiency into neurons and quantified STIM1 protein levels by immunofluorescent staining with the experimentalist blinded to sample identity (Fig. 4*F*). Consistent with our RT-qPCR and immunoblotting results, we observed a significant increase in STIM1 expression after *Neurod2* knock down (Fig. 4*G*,*H*). Importantly, we were able to rescue STIM1 levels by cotransfection of an shRNA-resistant mouse *Neurod2* cDNA, which was identical in its amino acid sequence to wild-type NEUROD2 (resND2; Fig. 4*E–H*). Finally, we also determined whether or not the mRNA level of *Stim2*, another sensor of ER Ca^2+^ and regulator of SOCE, was also affected after *Neurod2* suppression, and we observed a slight enrichment that was not statistically significant (1.5-fold enrichment; *p* > 0.05). Together, we concluded that NEUROD2 binding to the *Stim1* intronic element is correlated with a NEUROD2-dependent decrease in *Stim1* expression.

**Figure 5.**
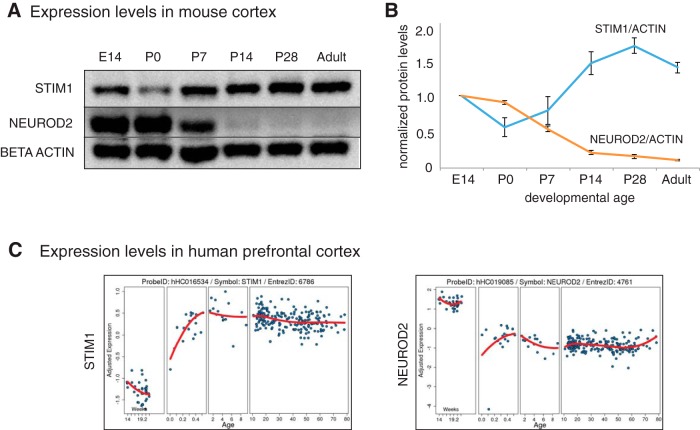
NEUROD2 and STIM1 expression are inversely correlated across cortical development. ***A***, ***B***, Immunoblotting analysis and quantification of protein levels across development in cerebral cortical tissue revealed an inverse correlation between NEUROD2 and STIM1 protein expression. STIM1 protein levels are normalized to the amount of β-actin. Data represents two biological replicates, each quantified as duplicates. Bars represent the SEM. ***C***, *Stim1* and *Neurod2* mRNA levels were plotted as a function of age in human prefrontal cortex using postmortem tissue. Plots were acquired from braincloud.jhmi.edu (Colantuoni et al., 2011). Similar to mouse data, an inverse correlation in *Neurod2* and *Stim1* expression was observed in humans as well.

Based on our results, we argued that a model in which NEUROD2 limited *Stim1* expression would be consistent with an inverse correlation in the levels of these two proteins across cortical development. Therefore, we determined NEUROD2 and STIM1 protein levels in various developmental ages in cortical tissue in mice (Fig. 5*A*). Our results demonstrated that, throughout development, a downregulation of NEUROD2 was correlated with an upregulation of STIM1 levels (Fig. 5*A*,*B*). Interestingly, a similar inverse correlation between *Neurod2* and *Stim1* mRNA levels was observed in prefrontal cortical tissue obtained across the human lifespan (Fig. 5*C*; braincloud.jhmi.edu/; Colantuoni et al., 2011). Together, our results are consistent with NEUROD2 acting as a brake on STIM1 expression in cortical neurons during development. However, since *Neurod2* is expressed only in excitatory neurons and *Stim1* is expressed in progenitors, neurons, as well as astrocytes (Kraft, 2015), the protein levels quantified by bulk analysis of cortical tissue reflected cumulative STIM1 levels from all these cell types. Future experimentation will help to clarify cell type-specific regulation of *Stim1* expression by NEUROD2 across cortical development.

### NEUROD2 is required for fine-tuning of SOCE in cortical neurons

**Figure 6.**
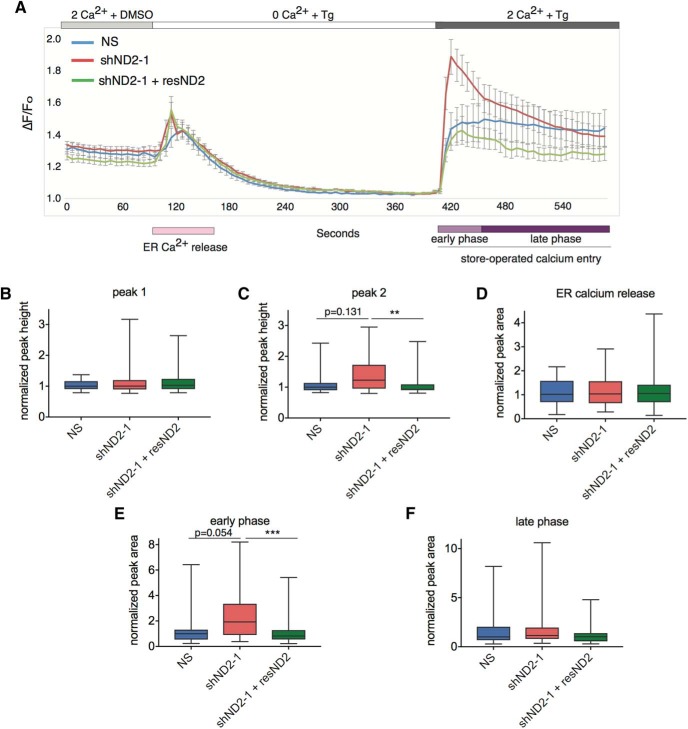
Suppression of *Neurod2* expression results in increased SOCE response. ***A***, Primary cortical cultures were transfected with NS shRNA or shND2-1 together with either an empty or shRNA-resistant *Neurod2* (resND2) expressing pcDNA4 vector. On the day of imaging, cultures were loaded with calcium-sensitive dye Fluo-3 and imaged by live imaging. Baseline signal was acquired by bathing the cells in Ringer’s buffer containing 2 µm Ca^2+^. Upon treatment with thapsigargin (Tg) and withdrawal of extracellular Ca^2+^, a first wave of rise in signal was observed that corresponded to emptying of ER Ca^2+^ stores (at ∼100 s). A second wave of signal was observed on providing Ca^2+^ containing Ringer’s buffer that corresponded to store-operated calcium entry (at ∼400 s). ***B***, ***C***, Quantification of initial peak heights for first wave (ER Ca^2+^ release) and second wave (SOCE) of Ca^2+^ signals unveiled an increase in SOCE on *Neurod2* knockdown that was rescued by coexpression of resND2. ***D–F***, Measurement of the total area under the peaks revealed that ER Ca^2+^ release was not affected; however, the early phase (∼50 s) but not the late phase of SOCE was significantly upregulated upon *Neurod2* suppression. Traces are color coded as follows: NS shRNA (blue); shND2-1 (red); and shND2-1 + resND2 (green). Data are presented as bar graphs; the line marks the median; the box represents the 25th and 75th percentiles; top and bottom whiskers mark minima and maxima, respectively. *Neurod2* is abbreviated as ND2. Nonparametric Kruskal–Wallis test was followed by Dunn’s multiple-comparison analysis, **p = 0.0073, ***p = 0.0008 (Table 2).

SOCE constitutes an important source of calcium entry and signaling in neurons. Briefly, depletion of ER Ca^2+^ stores causes the ER Ca^2+^ sensor STIM proteins (STIM 1 and STIM2) to interact with and activate cell surface Ca^2+^ release-activated Ca^2+^ (CRAC) channels, thereby resulting in a second wave of cytoplasmic Ca^2+^ rise (Moccia et al., 2015). Genetic suppression of *Stim1* or CRAC channels, or pharmacological blockade of CRAC channels, results in complete abrogation of this second wave of calcium rise that constitutes SOCE (Somasundaram et al., 2014). Since our data suggested that knockdown of *Neurod2* caused an upregulation in STIM1 levels, we next asked whether this effect also translated into deranged neuronal SOCE. We transfected primary cortical neurons with either NS shRNA or shND2-1 and measured cytoplasmic Ca^2+^ levels with the Ca^2+^-sensitive dye Fluo-3. We imaged transfected neurons selected by the coexpression of the marker gene mCherry. ER Ca^2+^ stores were released by treatment with 5 µm thapsigargin, a blocker of SERCA (sarcoplasmic/endoplasmic reticulum Ca^2+^ ATPase) pump. As expected, on thapsigargin treatment of neurons bathed in Ca^2+^-free buffer, we observed an initial wave of cytoplasmic Ca^2+^ increase both in control and NEUROD2-depleted neurons at comparable levels (Fig. 6*A*,*B*,*D*). As long as neurons were kept in Ca^2+^-free buffer, the ER stores remained empty, a situation that was presumably sensed by the Ca^2+^ sensor STIM1. On switching to a buffer containing 2 mM Ca^2+^, an immediate SOCE response was observed as a second wave of cytoplasmic Ca^2+^ rise. Consistent with a role for NEUROD2 in *Stim1* regulation, we observed an upregulation of SOCE in neurons depleted of NEUROD2, a response that was rescued by coexpression of shRNA-resistant resND2 (Fig. 6*A*,*C*,*E*). Specifically, on induction of SOCE, a significant upregulation of Ca^2+^ influx above control levels was detected during the early time periods (∼50 s) following NEUROD2 depletion, although this difference was attenuated during the later phase of SOCE (Fig. 6*E*,*F*).

**Figure 7.**
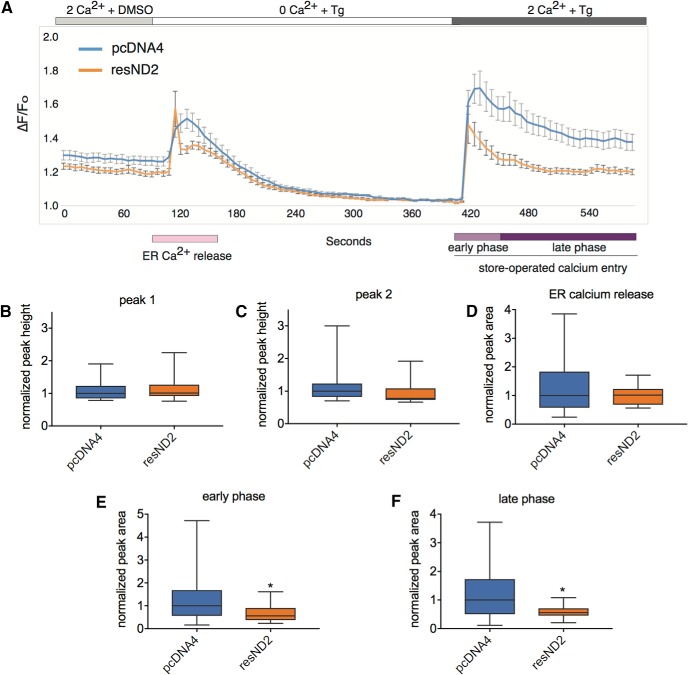
Overexpression of *Neurod2* reduces the SOCE response. ***A***, Primary cortical neurons were transfected with either empty or resND2 expressing pcDNA4 vector, and calcium imaging was performed as described in Figure 6*A*. ***B***, ***C***, Measurement of peak heights of first- and second-wave of Ca^2+^ signals revealed that overexpression of *Neurod2* in otherwise wild-type neurons causes a suppression of SOCE but does not affect steady-state levels of ER Ca^2+^. ***D–F***, Calculation of total area under peaks demonstrated that both late and early phases of SOCE are downregulated upon *Neurod2* overexpression. Traces are color coded as follows: pcDNA4 (blue) and resND2 (orange). Bars represent the SEM. Unpaired *t* test determined the p value: *p < 0.05. Data are presented as bar graphs; the line marks the median; the box represents the 25th and 75th percentiles; top and bottom whiskers mark minima and maxima, respectively (Table 2). ND2, *Neurod2*.

Next we tested the effects of *Neurod2* overexpression on SOCE response. We transfected primary cortical neurons with an empty vector or a vector overexpressing resND2 in otherwise wild-type neurons. In agreement with our prediction, we observed a significant suppression of SOCE (Fig. 7*A*,*E*,*F*) but not of steady-state ER Ca^2+^ levels (Fig. 7*A*,*B*,*D*) in neurons overexpressing NEUROD2 protein. Together, our results suggest that NEUROD2 fine-tunes neuronal SOCE by inhibiting STIM1 expression and that the level of SOCE response is regulated by the amount of NEUROD2 protein available in the cell.

## Discussion

### Implications of NEUROD2-regulated store-operated calcium entry

Almost all phases of cortical development from neural progenitor proliferation to axon guidance and synaptic plasticity are regulated by a range of calcium signaling pathways activated by different sources of calcium influx, including intracellular stores and SOCE (Uhlén et al., 2015). In contrast to Ca^2+^ influx through voltage- and neurotransmitter-gated calcium channels, little is known about how SOCE influences neuronal differentiation during cortical development. Accumulating evidence indicates that SOCE exists in neurons, where it is linked to neuronal growth cone turning, axon guidance, synaptic plasticity, and regulation of neuronal excitability (Park et al., 2010; Kraft, 2015; Majewski and Kuznicki, 2015; Moccia et al., 2015). However, how SOCE itself is regulated in neurons, which cellular components are involved, how they interact with different signaling pathways, and the nature of neuronal gene expression programs that establish and control SOCE are poorly understood. Our genome-wide target gene analysis has identified an unanticipated role for NEUROD2 during early postnatal cortical development as a suppressor of SOCE. Our results reveal that NEUROD2 binds to the second intron of the *Stim1* gene, and this association is linked to a decrease in *Stim1* expression. We have demonstrated that while *Neurod2* knockdown causes an increased SOCE response, its overexpression results in repressed SOCE. However, whether *Stim1* is the sole NEUROD2 target or whether additional target genes that control SOCE exist remains to be determined.

STIM proteins (STIM1 and STIM2) are ER Ca^2+^ store sensors that interact with plasma membrane-localized CRAC channels on ER Ca^2+^ depletion, inducing their opening and providing a subsequent wave of Ca^2+^ influx to refill the ER (Liou et al., 2005; Roos et al., 2005; Zhang et al., 2005). SOCE and specifically, the roles of Stim genes (*Stim1* and *Stim2*) are increasingly gaining attention as critical regulators of neuronal Ca^2+^ signaling and homeostasis. Recently, it was demonstrated that *Stim1* regulates axonal branching, growth cone guidance, cell migration, and neural progenitor cell proliferation (Mitchell et al., 2012; Shim et al., 2013; Somasundaram et al., 2014; Tsai et al., 2014). In particular, it appears that SOCE is tightly regulated within spatiotemporally confined subcellular domains, at least in part by the localization of STIM1 to compartments, such as the growth cones of pathfinding axons. It is noteworthy to mention that a downregulation of SOCE and upregulation of voltage-gated Ca^2+^ influx is observed as neural progenitors mature toward a neuronal fate within the developing cortex (Maric et al., 2000; D'Ascenzo et al., 2006; Somasundaram et al., 2014). The functional implications for this switch in the mode of calcium influx are currently unknown. The acquisition of an excitable membrane might allow neurons to activate additional signaling pathways, such as those activated by voltage-gated Ca^2+^ channels (VGCCs), that would be inaccessible in other cell types. Since the initiation of *Neurod2* expression also overlaps with neural progenitor cell cycle exit and differentiation to post-mitotic neurons, it will be interesting to explore whether and how NEUROD2 controls the transition from SOCE-mediated to voltage-mediated Ca^2+^ influx. Based on our results, we can sufficiently conclude that NEUROD2 is required for limiting *Stim1* expression in postmitotic neurons; however, future experiments will be required to understand the exact stage of neuronal differentiation in which this NEUROD2-dependent effect is initially observed.

Several studies have demonstrated STIM1-dependent reciprocal regulation between SOCE- and depolarization-induced Ca^2+^ influx. While the inhibition of *Stim1* caused a decrease in SOCE response, as expected, it also induced augmentation of Ca^2+^ influx through L-type VGCCs (Park et al., 2010; Wang et al., 2010). Interestingly, NEUROD2 transcriptional activity is induced on Ca^2+^ influx through VGCCs (Aizawa et al., 2004; Ince-Dunn et al., 2006). Together, one can imagine a scenario in which depolarization-induced calcium influx activates NEUROD2, which in turn calibrates levels of STIM1 and SOCE and, consequently, STIM1-dependent inhibition of L-type VGCCs.

To date, the role of STIM1 in radial migration of neurons in the developing cortex has not been investigated. However, a recent study investigating sheet migration of endothelial cells (human umbilical vein endothelial cells) demonstrate that STIM1 localizes to the leading edges of migrating cells (Tsai et al., 2014). Further gain-of-function and loss-of-function experiments in this study support a model where STIM1 is a negative regulator of migration speed. Interestingly, NEUROD2 is highly expressed within the cortex during the peak of radial migration (Telley et al., 2016). Although, endothelial sheet migration and neuronal radial migration most likely differ substantially from each other, we speculate that NEUROD2 might promote migration by suppressing a repressor of migration. On the other hand, additional studies investigating STIM1 impact on cell migration speed using various cancer cell lines have reported opposite results, suggesting that STIM1 is an accelerator of migration speed (Yang et al., 2009; Chen et al., 2011). While the involvement of STIM1 in the migration process is evident, its cell type-specific effects necessitate further investigations in relevant tissue and developmental stages.

### NEUROD2 as a transcriptional regulator

Our data demonstrate that one of the most prominent NEUROD2 binding sites in the mouse genome maps to a conserved intronic element enriched in E-boxes within the *Stim1* gene. Further, we demonstrate that shRNA-mediated suppression of *Neurod2* expression is correlated with an upregulation of *Stim1* mRNA and protein abundance. Our results uncover a novel mode of gene expression regulation by NEUROD2. To date, NEUROD2 has been presumed to act only as a transcriptional activator. Interestingly, in equivalent tissue and developmental stages, this element is also bound by CTCF, a transcription factor associated with chromatin insulators. Collectively, our results are consistent with a model in which NEUROD2 can fine-tune gene expression by functioning as part of an insulator complex and preventing the promoter from spuriously associating with enhancers located outside of the insulated neighborhood. Consequently, a downregulation of NEUROD2 protein levels may render the *Stim1* promoter vulnerable to the influence of an enhancer that can now ectopically promote transcription. It is generally acknowledged that, while not all CTCF peaks function as insulators, a large majority of insulators are bound by CTCF (Hnisz et al., 2016). Therefore, whether this *Stim1* intronic element is functioning as an insulator is currently unknown. In future experiments, we are very interested in further pursuing this possibility and testing whether this intronic element is required for limiting *Stim1* gene expression in a NEUROD2-dependent manner in neurons.

Our findings add to a series of recent discoveries of several neuronally expressed TFs limiting gene expression by binding to intronic elements (Wiegreffe et al., 2015; Rannals et al., 2016; Soldner et al., 2016). Most relevant to our findings is a study exploring TCF4, a ubiquitously expressed bHLH factor known to heterodimerize with tissue-specific bHLH TFs, including NEUROD2 (Ravanpay and Olson, 2008). The authors demonstrate that TCF4 binding to intronic elements within the *Scn10a* and *Kcnq1* genes, both encoding ion channels, induces a suppression of gene expression, which is associated with a deregulation of intrinsic excitability of cortical neurons (Rannals et al., 2016). In light of the finding that TCF4 biochemically interacts with NEUROD2 (Ravanpay and Olson, 2008), it is of future interest to test whether NEUROD2 and TCF4 functionally interact on specific sites to regulate gene expression.

In summary, our genome-wide target gene analysis of the neurogenic TF NEUROD2 has uncovered a novel gene expression mechanism by which differentiating cortical excitatory neurons fine-tune the extent of SOCE. We believe that future analyses of our results in the context of other high-throughput datasets representing TF binding, gene expression, chromatin accessibility, and conformation will provide valuable insights into how gene regulatory circuits function in guiding the differentiation of cortical excitatory neurons.

## Synthesis

Reviewing Editor: Orly Reiner, Weizmann Institute of Science Decisions are customarily a result of the Reviewing Editor and the peer reviewers coming together and discussing their recommendations until a consensus is reached. When revisions are invited, a fact-based synthesis statement explaining their decision and outlining what is needed to prepare a revision will be listed below. The following reviewer(s) agreed to reveal their identity: Jean-Bernard Manent, Takeshi Kawauchi.

Overall the reviewers have found that your study is interesting and worthy. They note that this is an interesting study suggesting that NeuroD2 may contribute to regulating calcium signaling and homeostasis in maturing neurons through controlling expression of the Stim1 gene, which encodes an ER calcium sensor.

However, they do find that several issues need to be addressed prior to publication, they believe that their comments can be resolved within two months of work. Their specific comments are summarized below:

Reviewer 1

In this study, the authors suggest that NeuroD2 may regulate expression of the Stim1 gene, which encodes an ER calcium sensor essential for controlling store-operated calcium entry. These are interesting novel findings, although several comments have to be addressed and additional evidences provided, especially for addressing comments #2 and #3.

1) From the provided material and text (‘we selected one of the highest scoring NEUROD2 binding sites’ pg15 ln7), it is unclear why the authors decided to focus their study on the second intron of the Stim1 gene among many other potential binding sites. What were the criteria and scoring method? How was the scoring cutoff defined? Etc. Clarification would help the reader a lot, and may be accompanied with a table showing other genes/binding sites.

2) Because they claim that NEUROD2 acts as a transcriptional repressor on Stim1 expression through binding E-box elements on intronic sequences of the Stim1 gene, the authors may wish to provide evidences that altering the sequence of these E-boxes (or removing them) results in increased Stim1 expression. If feasible, this would provide with more direct evidences than those of the NeuroD2 knockdown experiments.

3) Conclusions of the NeuroD2 experiments are somewhat overstated (‘NEUROD2 binding to Stim1 intronic element is associated with suppression of gene expression’ pg17, ln15). From the conducted experiments, the authors can reasonably conclude than knocking down NeuroD2 results in increased Stim1 mRNA and protein levels, but could hardly claim that this effect is a direct consequence of a reduced binding to the Stim1 gene intronic element (‘we concluded that NEUROD2 binding to the Stim1 intronic element is associated with a NEUROD2-dependent suppression of Stim1 expression’, pg 18 ln20-21). Direct evidences such as those suggested in the above comment are missing.

4) There is a general trend toward improving data representation in the scientific literature and Neurosciences so that distributional information regarding individual data points is available for the reader, especially for small samples. Distributional differences between conditions would be much more convincing if boxplots or scatterplots are utilized in the figures, especially for the Ca imaging experiments, as suggested in (Allen et al., Neuron 2012; Weissgerber et al., Plos Biol 2015).

5) The authors may wish to cite and discuss the results of a recent study utilizing a similar ChIP-seq analysis of NeuroD2 targets from murine embryonic neocortex (Bayam et al BMC Genomics 2015). Their results are complementary to those of the Bayam et al study.

Reviewer 2

The manuscript describes an upstream regulator for store-operated calcium entry (SOCE). The authors show that NeuroD2, a bHLH transcription factor, negatively regulates the expression of Stim1, a major ER calcium sensor, via the direct binding to the intron 2 of a Stim1 gene. They also show that knockdown or overexpression of NeuroD2 increases or decreases SOCE response, respectively. This study also provides evidence that NeuroD2 functions as a transcriptional repressor (in addition to an activator as previously described). The findings described in this manuscript are informative to many developmental neuroscientists, but there are several issues that should be improved.

[Major points]

1) Neuronal differentiation occurs in a stepwise fashion and a developing brain (e.g. E14.5 cerebral cortex) contains cells with several differentiation stages, including radial glial progenitors (Pax6+), intermediate progenitors (Tbr1+) and newly generated neurons (Tuj1+). As the authors mentioned, SOCE is downregulated in a subset of neuron-committed progenitors, but it is unclear whether these progenitors are Neurogenin-positive or NeuroD-positive or Tbr2-positive. To clarify it, immunohistochemistry experiments for NeuroD2 and Stim1 (and markers for neural progenitors or neurons) in the developing cerebral cortex are required.

2) It is strange that the culture periods are different in Fig. 4A-C (4 div), Fig. 4D-F (5 div) and Figs. 6 and 7 (7-9 div). The authors should consolidate the same culture period in all experiments. If there is a special reason, they should explain it and provide data of the expression levels of NeuroD2 and Stim1 in each time point. Particularly, the experiments in Figs. 6 and 7 should use the same stage of primary neurons.

3) In Fig. 4A-C, shRNAs were transfected immediately prior to plating, which may lead to defects in neuronal differentiation. Do these cells exhibit normal morphologies? The representative images are required. If neurite length would be short in the knockdown cells, actin (or tubulin) would not be appropriate for the loading control.

4) Neurons in the lower panels of Fig. 4E do not seem to be healthy. They do not extend their neurites.

[Minor points]

5) Student's t-test is not appropriate for statistical analyses of rescue experiments in Figs. 4F and 6B-F. Multiple comparison analyses should be used.

6) No error bars in Fig. 5B.

7) In Fig. 7, wild type-NeuroD2, but not rNeuroD2, should be used.
